# Prescribing Safety and Clinical Monitoring in Alcohol and Opioid Use Disorder: A Dual-Cycle Audit in a Mandated Rehabilitation Center in Qatar

**DOI:** 10.1192/bjo.2026.11852

**Published:** 2026-06-30

**Authors:** Ahmad Maaen Aalater, Nirvana Swamy Kudlur Chandrappa, Suhair Mohammed Yousuf, Dina ElGhar, Zeeshan Aqeel Usman Sheikh

**Affiliations:** Mental Health Services - HMC, Doha, Qatar

## Abstract

**Aims:**

Authors: Dr. Ahmad Maaen Aalater, Dr. Nirvana Swamy Kudlur Chandrappa, Dr. Faycal Walid Ikhlef, Dr. Suhair Mohammed Yousuf. Dr. Dina ElGhar, Dr. Zeeshan Aqeel Usman Sheikh, Dr. Majid Al-Abdulla,

To evaluate and compare prescribing patterns, safety practices, and clinical monitoring for patients with Alcohol Use Disorder (AUD) and Opioid Use Disorder (OUD) patients across two audit cycles at Umm Slal Treatment and Rehabilitation Center (USTRC) and to identify areas for sustained quality improvement.

**Methods:**

Two retrospective audits were conducted using inpatient records from 89 patients in 2022 and 79 patients in 2024 diagnosed with AUD or OUD. Extracted variables included medication indications, dose titration and adjustment, contraindications, adverse drug reactions (ADRs), drug-drug interactions, and completion of baseline investigations. The 2024 cycle was benchmarked against the prior audit to assess changes in prescribing safety and documentation.

**Results:**

In 2024, 46 patients had AUD and 19 had OUD, with most patients aged 20–29 years. Documentation of appropriate medication indications improved (AUD 72%; OUD 78.9%). Dose titration and dose adjustment remained consistently high (98–100%), and contraindications were appropriately avoided in nearly all cases. Baseline medical testing improved but remained incomplete, with 8.7% of AUD and 10.5% of OUD patients missing liver or renal function results. Adverse Drug Reactions (ADRs) were documented in 18% of AUD and 5.3% of OUD cases, predominantly extrapyramidal symptoms and sedation. Drug interactions were identified in approximately one-third of prescriptions, with 25-29% classified as higher-risk interactions requiring closer monitoring. Documentation gaps and limited multidisciplinary ADR reporting persisted across cycles.

**Conclusion:**

As one of the first longitudinal prescribing-safety audits within a mandated addiction treatment service in the region, this study demonstrates strong adherence to dosing safety while identifying ongoing vulnerabilities in documentation quality, baseline testing, and interaction monitoring. Sustained staff education, structured electronic documentation fields, and continuous re-audit are essential to strengthening medication safety and supporting high-quality addiction care in compulsory treatment settings.

